# Breaking bad news in ophthalmology: a literature review

**DOI:** 10.5935/0004-2749.2022-0104

**Published:** 2022-09-06

**Authors:** Cecília Francini Cabral de Vasconcellos, Marina Cruz Pellissari, Olivia Araujo Zin, Mariana Valim Salles, Juliana Maria Ferraz Sallum, José Paulo Cabral de Vasconcellos

**Affiliations:** 1 Department of Ophthalmology, Escola Paulista de Medicina, Universidade Federal de São Paulo, São Paulo, SP, Brazil; 2 Department of Ophthalmology, Universidade de Campinas, Campinas, SP, Brazil

**Keywords:** Breaking bad news, Communication, Clinical Competence, Physician-patient relationship, Ophthalmology, Truth disclosure, Comunicação de más notícias, Comunicação, Competência Clínica, Relações médico-paciente, Oftalmologia, Revelação da verdade

## Abstract

Medical specialties have recognized that breaking bad news assists clinical practice by mitigating the impact of difficult conversations. This scenario also encourages various studies on breaking bad news in ophthalmology since certain ocular diagnoses can be considered bad news. Thus, the objective is to review the scientific literature on breaking bad news in ophthalmology. The literature databases like MEDLINE/PUBMED, EMBASE, LILACS, SCOPUS, COCHRANE, and SCIELO, were screened for related research publications. Two independent reviewers read all the articles and short-listed the most relevant ones. Seven articles, in the formats of original article, review, editorial, oral communication, and correspondence, were reviewed. Conclusively it reveals that ophthalmologists are concerned with communicating bad news effectively but lack related studies. Nevertheless, there is a growing realization that training in breaking bad news can increase physicians’ confidence during communication, thus, benefiting the therapeutic relationship with the patient and his family. Therefore, it would be valuable to include breaking bad news training in the curriculum of residencies.

## Abstract (pt)

O reconhecimento sobre a comunicação de más notícias como mitigadora de conversas difíceis por outras especialidades médicas, incentiva o estudo desta temática na oftalmologia. Sendo assim, o objetivo deste estudo é revisar a produção de pesquisas científicas sobre a comunicação de más notícias em oftalmologia. Para isso, foi realizada uma revisão de literatura. As bases de dados utilizadas foram MEDLINE/PUBMED, EMBASE, LILACS, SCOPUS, COCHRANE e SCIELO. Dois revisores independentes leram todos os artigos e selecionaram a amostra final. Sete artigos foram escolhidos nos formatos de artigo original, revisão, editorial, comunicação oral e correspondência. Os oftalmologistas estão preocupados em comunicar as más notícias de forma eficaz, mas faltam estudos sobre o tema. No entanto, há uma crescente percepção de que o treinamento de comunicação de más notícias aumenta a confiança dos médicos na comunicação, beneficiando a relação terapêutica. Portanto, seria valioso incluir este treinamento no currículo das residências.

## INTRODUCTION

Breaking bad news (BBN) is the communication of critical health issues when patients experience a drastic negative change in their current reality and future perspective^(1)^. It is usually performed by healthcare professionals and directly impacts the patients’ experience with the health/disease process and prognosis^(2,3)^.

In medical specialties such as oncology and palliative care, the BBN process is an important part of the treatment, requiring an in-depth study of BBN’s biop-sychosocial impacts^(4,5)^. In other clinical specialties that address genetic, hereditary, and degenerative processes, bad news refers to the lack of effective treatment and worsening of the clinical condition.

In ophthalmology, several diagnoses can be considered critical or bad news, such as glaucoma, inherited retinal dystrophies, age-related macular degeneration, and ocular trauma^(6, 7, 8)^. Many studies have demonstrated that eye diseases that cause considerable loss of vision lead to a significant reduction in patients’ quality of life and increase comorbidities, such as anxiety and depression^(9^, ^10, 11)^.

Even though it is not life-threatening, visual loss is experienced as the death of one of the senses and drastically reduce the level of autonomy and performance of daily activities. Families also experience changes in their routines as they have to accompany the patient everywhere^(11, 12^, ^13)^.

Due to the particularities listed above, communication of bad news can be considered a delicate process by medical teams and of great importance in the adaptation of the patient to the disease^(14,15)^. Therefore, training protocols were created to guide BBN.

Several medical residences train their professionals for effective and adequate communication of bad news. Training can take place through expository classes, roleplay, and guided practices based on protocols^(16,17)^. Internationally, the most used protocol is the SPIKES protocol created by Buckman in 1992. Terminal illnesses and grieving processes have been attenuated through such protocols^(18,19)^.

SPIKES is an acronym, and each letter represents an orientation step in doctor/patient communication.

- Setting up: The induction point of the relationship, must be conducted in an accessible and welcoming manner.
- Perception: The time to understand the patient’s knowledge about diagnosis and possible prognosis.
- Invitation: The specialist/healthcare provider invites the patient to discuss the illness and to determine the depth of the discussion. This determination is based on the patient’s desire and the understanding of the cognitive and emotional condition.
- Knowledge: It is the passing of information in an accessible and personalized way, leading to an emotional reaction to the news.
- Emotions: The moment of emotional reaction to the news; must be treated with sensitivity, acceptance, and empathy.
- Strategy and Summary: It is the time to make necessary referrals, assessing the biopsychosocial conditions and the patient’s diagnosis^(18,19)^.

In 1991, the Institute for Families of Blind Children produced the first documentary related to BBN in ophthalmology. This documentary demonstrated the importance of proper BBN in ophthalmology and how unprepared professionals could lead to difficult conversations^(20)^.

Medical specialties such as oncology and palliative care have recognized that BBN assists clinical practice by mitigating the impact of difficult conversations. This scenario encourages the expansion of studies on BBN in ophthalmology since certain ocular diagnoses can be considered bad news. The objective of this study is to review scientific research on BBN in ophthalmology, explicating current knowledge in this area and suggesting points to be evaluated in the future.

## METHODS

We conducted a literature review using our registered account in the Open Science Framework (https://osf.io/chdzy/).

The inclusion criteria for the articles were as follows: articles that addressed BBN in ophthalmology in different formats, such as original article, review, editorial, oral communication, and correspondence.

The exclusion criteria for the articles were as follows: articles written in a language other than English, Spanish, Portuguese, and French and replicated articles.

The first phase involved the search for keywords related to the research question in different databases, namely, MEDLINE/PUBMED, LILACS, SCOPUS, EMBASE, COCHRANE, and SCIELO. The key words searched included Breaking Bad News * AND Ophthalmology, Breaking Bad News * AND Eye Disease, Breaking Bad News *AND Ocular disease, Truth Disclosure * AND Ophthalmology, Truth Disclosure * AND Eye Disease, Truth Disclosure * AND Ocular Disease, difficult conversation * AND Ophthalmology, difficult conversation * AND Eye Disease, difficult conversation *AND Ocular disease.

Two independent reviewers with a degree in psychology read the titles and abstracts and selected the articles for initial sampling. Then, based on the inclusion and exclusion criteria, reviewers determined the articles in the final sample and read them in full. Data extracted from the selected articles were first author, year of publication, title, country of origin, type of article, main points discussed, and the conclusions.

## RESULTS

**Figure 1:**
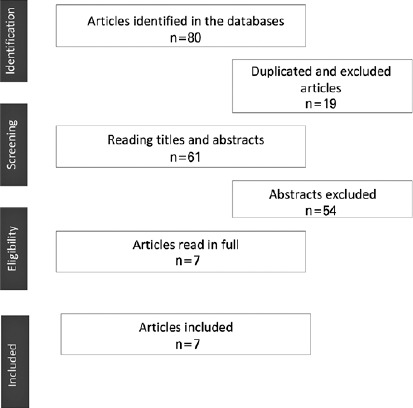
Depiction of the literature review fowchart

Screening of various literature databases yielded 80 related articles, of which 61 articles were without repetition. On the basis of the inclusion criteria, seven articles were read in full and selected for the final sample. A flowchart depicting literature screening process and its results is provided (Figure 1).

The selected articles have the following formats: editorial, communication, mini-review, correspondence, and original article. The selected articles originated from only four (4) countries: two (2) from North America (United States of America (USA) and Canada) and two (2) from Europe (France and the United Kingdom). Table 1 shows the main data for each included article.

**Table 1:** Main data of the included articles

Premier author	Year of publication	Title	Countries	Type of article
Vander VeenDK^(21)^	2019	An ophthalmologist’s view on breaking bad news to patients	United States of America	Editorial
Mancel Salino EH^(22)^	2009	Approche culturelle dans l’annonce du diagnostic en ophtalmologie [Cultural approach of the diagnosis announcement in ophthalmology]	France	Oral Communication
Hilkert SM^(23)^	2016	Breaking bad news: a communication competency for ophthalmology training programs	United States of America	Original Article
Genies P^(24)^	2009	Comment annoncer le glaucome à un patient ? [How do you tell a patient about glaucoma?]	France	Mini-Review
Anderson MF^(25)^	2010	Diagnostic information provided by referrers to patients with suspected uveal melanoma	United Kingdom	Correspondence
Zakrzewski PA^(26)^	2008	Should ophthalmologists receive communication skills training in breaking bad news?	Canada	Original Article
Mishra A^(27)^	2017	Communication Skills Training in Ophthalmology: Results of a Needs Assessment and Pilot Training Program	United States of America and Canada	Original Article

### Main points and conclusions of the articles

VanderVeen^(21)^ commented on syndromic ophthalmic cases that require multidisciplinary teams and are increasingly diagnosed through genetic testing, citing a disease called neuronal ceroid lipofuscinosis. The main conclusions referred to patience and time needed to deal with difficult conversations with the need for sincerity in the diagnosis and a simplified explanation.

The main objective of these conversations was to make patients and families feel that there will always be something needed to be done and that the medical teams will take responsibility for the case.

The intercultural approach was discussed by Mancel Salino^(22)^ through a case report on the announcement of childhood blindness, exploring the importance of culture in the understanding of a disease. The three key points for the intercultural approach included the knowledge of the meaning of health/illness for a certain culture, the integration of this knowledge during consultations, and the mediation of elements of the culture during crises. This approach leads to greater physician ease during difficult conversations and patients’ understanding of their diagnoses and prognoses.

Hilkert et al.^(23)^ pointed out protocols and training for BBN in various medical specialties, designing and evaluating a BBN pilot training in ophthalmology. According to the results, ophthalmologists (both teachers and residents) perceived the need for structured BBN training, with 73% indicated the need during residency. The training demonstrated an improvement in residents’ confidence while communicating the bad news and their willingness to use the SPIKES protocol.

Glaucoma is a chronic disease that requires daily treatment, and Genies^(24)^ suggested tips on how to convey its diagnosis. Communication should be simple and frank, demonstrating the importance of adherence to the treatment. The key points raised by the author were: assessment of the doctor’s personality and the patient’s personality, attention to the past experiences lived by the patient as well as an understanding of the disease as limiting vision and quality of life. Therefore, appropriate communication benefits the therapeutic relationship and increases the patient’s adherence to the treatment.

The correspondence by Anderson et al.^(25)^ discusses the difficulty in communicating the diagnosis of uveal melanoma. A retrospective survey conducted at an ocular oncology center assessed whether patients were informed about their diagnosis before referral to oncology and how they felt about this communication. Sixty-four percent of the patients stated that they had been informed of their malignancy by the referring physician. Moreover, the same number of patients preferred their referrer to be the person to communicate about their diagnoses. According to the patients, receiving proper information about the diagnosis before going to an oncology center could increase comfort and readiness during the appointment.

Zakrzewski et al.^(26)^ assessed whether ophthalmologists perceived the importance of formal BBN training. The survey was conducted through an online form with the Canadian ophthalmology community. According to the results, ophthalmologists recognized the importance of adequate communication, and that such competence can be learned. Training should be done during residency and benefit the doctor-patient relationship. The authors also stated some ophthalmic situations that need to be considered bad news. Such situations are ocular/orbital malignancies, medical/surgical errors, pediatric eye diseases, need for enucleation, and disclosure and counseling about genetic eye diseases.

Mishra et al.^(27)^ investigated the perception of directors and residents about the need for BBN training. A nationwide survey was carried out with directors of ophthalmology residencies and residents from a specific program. BBN workshop was conducted upon completion of the study. Directors highlighted the need to improve their training and residents demonstrated difficulties managing their emotions during difficult conversations. The workshop improved the residents’ ability to manage emotions. According to the residents, the emotions experienced during difficult conversations are anxiety, frustration, empathy, exhaustion, and insecurity/sadness.

## DISCUSSION

Our review suggests a scarcity of studies on the communication of bad news for eye diseases. Existing studies are restricted to some developed countries, and most of them have emerged only in the last decade. In Latin America, there are no studies on this subject, making this the first review of its kind. The increase in research on this topic can demonstrate its importance in ophthalmological, clinical, and surgical practices^(20^, ^21, 22, 23, 24, 25)^.

VanderVeen^(21)^, in their reflection on BBN for the neuronal ceroid lipofuscinosis cases, emphasized that it is recommended to transmit knowledge and information to patients. However, one should be careful with speculations about the degree of disease progression and the duration for each loss to occur. Understanding the impossibility of quantifying and dating the evolution of disease is essential to avoid false expectations and unfounded hopes. This thought can be extended to other chronic and limiting illnesses apart from only a specific disease.

Mancel Salino^(22)^ investigated cultural differences and their impact on understanding eye diseases. Often, having a disease can mean a lack of self-care or difficulty to undergo appropriate treatments. The sensitivity of knowing the patient’s perspective and adjusting to that condition brings assiduity and confidence to the specialist/patient relationship.

Some of the requirements for effective communication of bad news are active listening, sensitivity, and the perception of others^(22)^.

- Active listening: During communication, we must be available to listen to others, free from judgment and preconceived ideas. Be present and attentive to intervene truly and consistently.
- Sensitivity: Understanding and paying attention to the emotions and sensations of others, being able to be empathetic to what others transmit. It is not necessary to feel for the other but to understand and respect their emotions.
- Perception of others: Perceiving others is not limited to physical observation but to understanding how much they expect from that encounter and what resources are available to receive the bad news^(22)^.

Communication of bad news can be practiced in different ways by different professionals, but it can also be learned through a theoretical and practical teaching process. Hilkert et al.^(23)^, and Zakrzewski et al.^(26)^, evaluated the opinion of ophthalmologists on the relevance of BBN training. The result concluded that BBN training would improve communication and should be included in the formal residency curriculum. Learning from appropriate and inappropriate care examples expands the repertoire of all professionals involved.

The pilot training program conducted by Hilkert et al.^(23)^ resulted in a significant increase in residents’ confidence in communicating bad news and setting realistic expectations without destroying patients’ hope. All evaluated residents agreed to use the SPIKES protocol during their clinical consultations.

The SPIKES protocol is easy to understand and has a flexible structure which facilitates its application in various medical specialties. The protocol guides how to approach the patient from the beginning of care until referral. Moreover, it does not emphasize any one specific disease.

Ginies^(24)^ and Anderson et al.^(25)^ reported that all communications involve two sides, the one who gives and the other who receives the news. Each participant in this process is a unique being with different biopsychosocial characteristics. For a meeting to be deemed satisfactory, there must be listening and an exchange of knowledge by each party. The specialist must contribute with their technical knowledge and patients with their experience and perception of the disease.

In an adequate specialist-patient relationship, technical knowledge about the characteristics and evolution of the disease must be added to the impact of the disease on the quality of life. This exchange allows a comprehensive view of individuals and how they live with their disease process. In addition, individuals must feel understood and invited to decision-making about their healthcare^(23,25)^.

Some ophthalmological diseases such as uveitis require continuous treatment that takes time and generates financial costs. These types of chronic eye diseases also require proper communication, as they will imply a drastic change in the routines of patients and family members. Referrals to rehabilitation or psychotherapy can also benefit patients as learning to deal with changes in routine and loss of vision makes it easier for patients to live with their disease^(28,29)^.

In the case of eye diseases without any effective treat ment available, referral to social and rehabilitation centers is recommended. It is observed that palliative care improves the quality of life and acceptance of chronic eye diseases by the patients and their families. Adult patients can be rehabilitated with orientation and mobility training, activities of daily living, and assistive technologies. On the other hand, visually impaired children can go through a habilitation process with continuous monitoring of their cognitive, educational, and behavioral development^(21,23,24)^.

If medical care and referral to rehabilitation are not provided, or patients fail to understand the importance of specialized care, then they may have difficulty in conceiving their new reality and rely solely with the expectation of cure or treatment. In addition, patients may not be able to create coping strategies without assistance and may feel sad, anxious, and inadequate. In such situations, what is at stake is not the importance of scientific advancement, but how patients live their lives while treatments are not available^(21,22,24)^. Therefore, more studies are needed to incorporate BBN in the field of ophthalmology and systematize referrals for rehabilitation.

The limitations of this study are the failure to assess the methodological quality of the reviewed articles and the non-statistical assessment of the results in the included studies.

Creating a welcoming environment and following the demands of active listening, sensitivity, and perception of others can help the healthcare providers during the treatment process. In addition, being aware of the biopsychosocial impact of the disease, and referring patients to rehabilitation, corroborates a humanized and interdisciplinary BBN.

Ophthalmologists are concerned with communicating bad news effectively, but there are not many studies on this topic. However, there is a growing realization that BBN training increases physician confidence in communication and benefits the therapeutic relationship. We thus suggest here that BBN training must be included in the formal curriculum of residencies along with the use of well-established protocols such as SPIKES.
